# Survey of the Endophytic Bacteria Inhabiting Wild *Daucus* Seed Using 16S rRNA Gene Amplicon Sequencing

**DOI:** 10.1128/mra.00140-23

**Published:** 2023-05-08

**Authors:** Sandeep Kumar, Linda J. Johnson, Suliana Teasdale, Yulia Morozova, Anouck C. M. de Bonth, Ruy Jauregui, Rina Hannaford, Stuart D. Card

**Affiliations:** a AgResearch Ltd., Grasslands Research Centre, Palmerston North, New Zealand; University of California, Riverside

## Abstract

We report a preliminary survey of the endophytic bacterial microbiota of seed from wild carrot (Daucus carota) using 16S rRNA gene amplicon sequencing. *Actinobacteria*, *Bacteroidetes*, *Firmicutes*, and *Proteobacteria* were the most abundant phyla detected, while *Bacillus*, *Massilia*, *Paenibacillus*, *Pantoea*, Pseudomonas, *Rhizobium*, *Sphingomonas*, and *Xanthomonas* were the most abundant genera.

## ANNOUNCEMENT

*Daucus* (family Apiaceae) is a genus of herbaceous plants that contains around 25 species, with the most recognized being Daucus carota subsp. *sativus*, the cultivated carrot. In terms of global yield, carrots are among the top vegetable crops, with annual production (together with turnips) of >40 million tons (1). As with many vascular plants, *Daucus* associates with endophytic microorganisms that can confer beneficial traits to the host (2). Many authors hypothesize that crop domestication, coupled with the practice of intensive agriculture, has reduced the abundance and diversity of microbiota that reside within plants, including their seed (3, 4). This study surveyed the bacterial endophytic communities of seeds harvested from 48 wild *Daucus* accessions, sourced mainly from New Zealand (Table 1).

**TABLE 1:** Details of *Daucus* samples used in this study and information on sequencing reads

Sample	Country of origin	Original accession name	No. of raw reads	No. of quality-filtered reads	SRA accession no.
WD01	New Zealand	Brookside	79,872	31,177	SRR23240437
WD02	New Zealand	Bulls	80,031	34,553	SRR23240436
WD03	New Zealand	Doyleston	79,955	40,958	SRR23240413
WD04	New Zealand	Eiffleton	80,000	30,368	SRR23240402
WD05	New Zealand	Feilding	79,767	30,004	SRR23240391
WD06	New Zealand	Rangiora	79,949	51,864	SRR23240426
WD07	New Zealand	Hororata	79,921	30,425	SRR23240425
WD08	New Zealand	Greenpark	80,189	29,630	SRR23240424
WD09	New Zealand	Hinds	80,173	72,232	SRR23240423
WD10	New Zealand	Irwell	79,912	37,097	SRR23240422
WD11	New Zealand	Kai Iwi	79,674	34,133	SRR23240435
WD12	New Zealand	Kaitoke	79,754	39,548	SRR23240434
WD13	New Zealand	Leeston	79,720	31,244	SRR23240421
WD14	New Zealand	Manutahi	79,652	30,041	SRR23240420
WD15	New Zealand	Maxwell	80,061	33,522	SRR23240419
WD16	New Zealand	Caldwells road	80,339	41,296	SRR23240418
WD17	New Zealand	Patea	80,031	26,251	SRR23240417
WD18	New Zealand	Sample A	80,182	32,147	SRR23240416
WD19	New Zealand	Selwyn riverbed	80,223	40,069	SRR23240415
WD20	New Zealand	T Base 2	79,766	49,943	SRR23240414
WD21	New Zealand	Turakina	79,809	31,016	SRR23240412
WD22	New Zealand	Wanganui	79,955	25,368	SRR23240411
WD23	Britain	1581004	79,940	57,886	SRR23240410
WD24	Ireland	1470441	79,888	60,550	SRR23240409
WD25	Netherlands	1581001	79,828	49,149	SRR23240408
WD26	Portugal	1581006	79,928	65,372	SRR23240407
WD27	Spain	1581007	80,161	50,940	SRR23240406
WD28	Uzbekistan	1580992	80,139	49,638	SRR23240405
WD29	Cyprus	DAU531	80,309	60,038	SRR23240404
WD30	France	K6670	80,140	57,742	SRR23240403
WD31	Germany	DAU361	80,136	47,115	SRR23240401
WD32	Greece	DAU239	71,870	53,370	SRR23240400
WD33	Italy	DAU486	79,863	60,427	SRR23240399
WD34	Portugal	DAU427	80,073	49,126	SRR23240398
WD35	Spain	DAU265	80,099	68,991	SRR23240397
WD36	Tunisia	DAU249	79,988	33,818	SRR23240396
WD37	Uzbekistan	DAU214	80,113	54,537	SRR23240395
WD38	Denmark	NGB21720	80,067	31,977	SRR23240394
WD39	Sweden	NGB16882	80,132	65,263	SRR23240393
WD40	Estonia	Ames 17826	80,349	67,997	SRR23240392
WD41	Israel	Pl 390884	80,198	54,800	SRR23240390
WD42	Israel	Pl 390881	80,164	64,911	SRR23240433
WD43	Kazakhstan	Pl 652213	79,875	62,176	SRR23240432
WD44	Libya	Pl 279764	79,590	67,040	SRR23240431
WD45	Poland	Pl 652192	80,108	16,910	SRR23240430
WD46	Poland	Pl 652190	80,061	48,888	SRR23240429
WD47	Turkey	Pl 305443	43,228	23,951	SRR23240428
WD48	Uruguay	Pl 287113	79,926	56,842	SRR23240427

To profile the bacterial diversity of wild *Daucus* seeds, 40 seeds from each accession were surface disinfected following the methods described by Beirinckx et al. (5). Seeds were germinated on 1% water agar at 22°C with a 16-h photoperiod. Twenty healthy seedlings (i.e., those free of any disease symptoms) were selected and ground in liquid nitrogen. Ground seedlings were processed with the DNeasy plant minikit (Qiagen GmbH, Hilden, Germany) according to the manufacturer’s instructions. A DNA library was prepared for the V3 to V4 region of the 16S rRNA gene using the primer pair 335F (5′-CADACTCCTACGGGAGGC-3′) and 769R (5′-ATCCTGTTTGMTMCCCVCRC-3′) (6) and then was sequenced (250-bp paired-end reads) using the MiSeq platform (Illumina Inc., San Diego, CA, USA). Paired-end sequence read data were processed using the QIIME v2 (7)-recommended DADA2 pipeline (8). Processed reads were used to construct the feature table at the amplicon sequence variant (ASV) level. The SILVA v132 database (9) was used for the taxonomic assignment of ASVs. The statistical analysis was accomplished using the R package phyloseq v1.34.0 (10) in R v4.0.2 (11) within RStudio v1.3.1093 (12).

**FIG 1:**
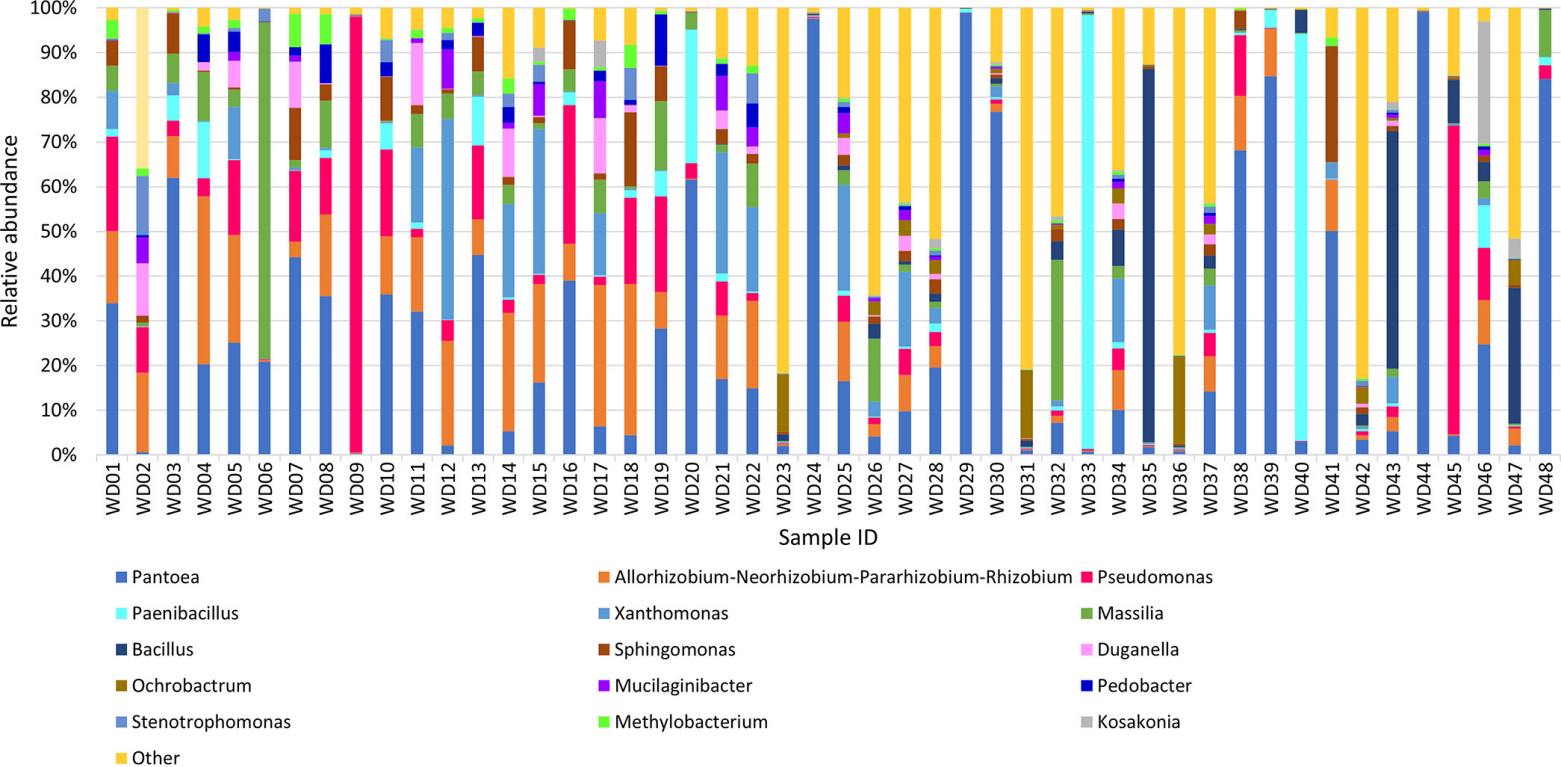
Bacterial community compositions in wild *Daucus* samples, showing the relative abundance of bacterial genera across seed accessions using taxonomic assignment of ASVs. The most abundant genera are listed, while genera with low average abundance (<1%) are combined as one group designated “Other.”

*Actinobacteria*, *Bacteroidetes*, *Firmicutes*, and *Proteobacteria* were the most abundant and prevalent phyla across all samples, covering >97% of the total reads. These phyla were previously reported as the most abundant microbiota among several different plant species, including *Brassicaceae* and *Poaceae* species (13, 14). *Bacillus*, *Massilia*, *Paenibacillus*, *Pantoea*, Pseudomonas, *Rhizobium*, *Sphingomonas*, and *Xanthomonas* were the most abundant and prevalent genera within our wild *Daucus* samples, with an average relative abundance varying from 25.7% to 3.1%. These genera covered >67% of the total reads (Fig. 1).

Few studies have investigated the seed microbiome of *Daucus* spp.; however, a culture-dependent study of the root microbiota of several carrot cultivars from Canada identified *Agrobacterium*, Pseudomonas, and Staphylococcus as the most abundant genera, with several strains of Pseudomonas fluorescens with growth-promoting traits being identified (15). Although several genera identified in our study contain pathogenic species, the seed microbiome can contain nonpathogenic relatives of these bacteria. For example, novel, nonpathogenic strains of *Xanthomonas* displaying strong bioprotection activities have been isolated from ryegrass seed (15). Future research will aim to determine whether the seeds of these wild accessions contain a greater abundance and/or diversity of microbes, compared with New Zealand-bred carrot cultivars.

### Data availability.

The 16S rRNA gene amplicon sequence data for the *Daucus* samples have been deposited in the GenBank Sequence Read Archive (SRA) under the BioProject accession number PRJNA928079.
